# Supplementary material for: AF Inducibility Is Related to Conduction Abnormalities at Bachmann’s Bundle
Source: J Clin Med. 2021 Nov 26;10(23):5536. doi: 10.3390/jcm10235536 (PMC8658171; doi:10.3390/jcm10235536)
Supplement: Supplementary file 1 [file jcm-10-05536-s001.zip › jcm-1467518-supplementary.pdf]

Supplementary Materials

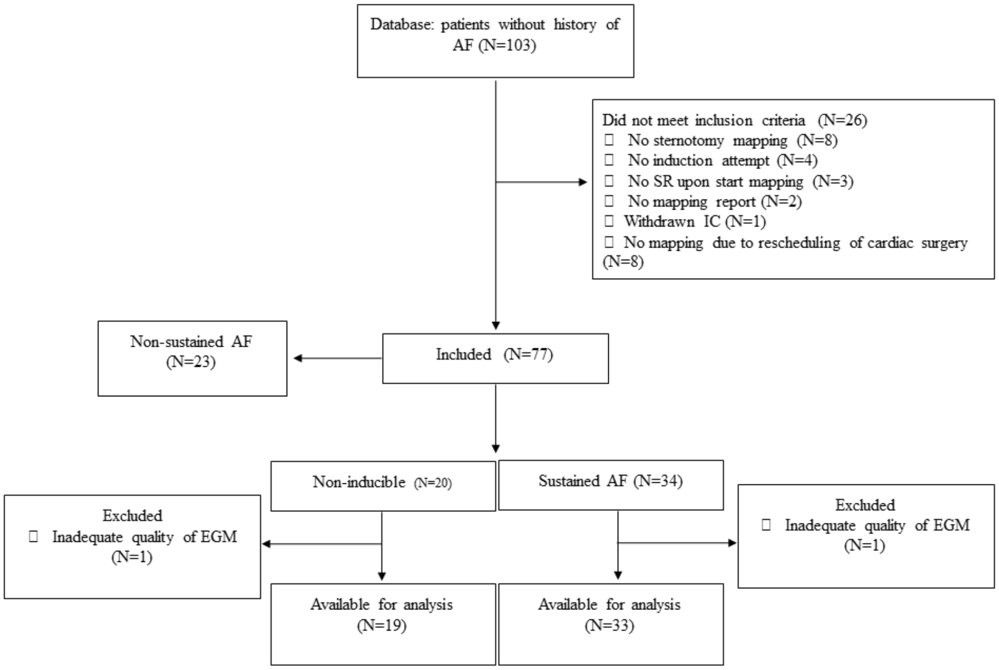

Figure S1: Flow Diagram of patient selection. This CONSORT flowchart demonstrates the data selection process and various reasons for patient exclusion. AF: atrial fibrillation, EGM: electrograms, IC: informed consent, SR: sinus rhythm.

Table S1. Univariate predictors for AF inducibility

Dependent: AF inducibility

Univariate variable	OR [95% CI]	*p*-value
LA dilatation	4.72 [1.16–19.3]	0.030
BMI	1.14 [0.99–1.33]	0.074
Maximum length of CB line	1.06 [1.00–1.11]	0.032
TAT	1.05 [1.01–1.10]	0.026
SimAct	4.2 [1.02–17.0]	0.047

OR = Odds Ratio, AF = atrial fibrillation, BMI = body mass index, CB= conduction block, LA = left atrium, SimAct = simultaneous activation, TAT = total activation time

Table S2. Multivariate predictors for AF inducibility

Dependent: AF inducibility

	**Corrected for LA dilatation**		**Corrected for BMI**		**Corrected for LA dilatation and BMI**	
Variables	OR [95% CI]	*p*-value	OR [95% CI]	*p*-value	OR [95% CI]	*p*-value
LA dilatation			5.55 [1.30–23.8]	0.021		
BMI	1.16 [1.00–1.36]	0.053				
Maximum length of CB line	1.01 [1.00–1.12]	0.028	1.15 [0.99–1.06]	0.056	1.06[1.00–1.12]	0.047
TAT	1.05 [1.00–1.11]	0.040	1.05 [1.00–1.10]	0.036	1.05[0.99–19.7]	0.065
SimAct	5.4 [1.23–24.1]	0.026	3.08 [0.70–13.7]	0.139	3.87[0.81–18.6]	0.091

OR = Odds Ratio, AF = atrial fibrillation, BMI = body mass index, CB = conduction block, LA = left atrium, SimAct = simultaneous activation, TAT = total activation time
